# Sexual dimorphism in the DNA methylation pattern after immune stimulation in the zebrafish (*Danio rerio*) gonads

**DOI:** 10.3389/fcell.2025.1720219

**Published:** 2025-12-19

**Authors:** M. Salazar, M. Caballero-Huertas, T. A. van Gelderen, M. N. Krabbe, M. Gut, S. Heath, A. Esteve, L. Ribas

**Affiliations:** 1 Institut de Ciències del Mar, Consejo Superior de Investigaciones Científicas (ICM-CSIC), Barcelona, Spain; 2 CIRAD, UMR, ISEM, Montpellier, France; 3 ISEM, Université de Montpellier, CNRS, IRD, CIRAD, Montpellier, France; 4 Centre Nacional de Anàlisis Genòmic (CNAG), Barcelona, Spain; 5 Universitat de Barcelona (UB), Barcelona, Spain

**Keywords:** sex ratio, immune, reproduction, aquaculture, epigenetics, early development, bacterial

## Abstract

**Introduction:**

In fish, epigenetic modifications are fundamental for regulating development, growth and adaptation to environmental factors. Emerging evidence further suggests that epigenetic mechanisms may modulate how fish gonads respond to infectious agents. Gonadal factors—including reproductive hormones and cytokines—are known to influence immune-cell activities, regulate the production of immune molecules, affect the overall immune response, and participate in gonadal sex differentiation. Although interactions between the reproductive and immune systems are well established, the epigenetic mechanisms underlying this interaction remain insufficiently elucidated, both in fish and in mammals. This study investigates how immune stimulation affects sex differentiation and methylation patterns of innate-immune genes in zebrafish gonads.

**Methods:**

To study the epigenetic events involved in the immune–reproduction interaction, zebrafish (*Danio rerio*) were immune-stimulated with lipopolysaccharide (LPS) using two experimental approaches. (1) To assess the effect of immune stimulation on sex ratio, larvae were bathed in LPS during gonadal development (17–30 days post-fertilization, dpf). (2) To examine DNA methylation patterns in response to immune stimulation in adulthood, sexually mature fish received intraperitoneal LPS injections. Methylation analyses focused on two key innate immune genes, *caspase 9* (*Casp9*) and *interleukin 1β* (*Il1β*). DNA methylation was quantified using a candidate-gene approach at single-nucleotide resolution through sequencing of bisulfite-converted DNA.

**Results:**

Immune stimulation during gonadal development did not produce a statistically significant difference in sex ratio, although a clear trend toward feminization was observed in LPS-treated fish. In adults, *Casp9* exhibited significant DNA-methylation differences driven by the interaction between treatment and sex. Specifically, eight CpG sites were significantly altered in treated females, while three CpG sites were significantly altered in treated males. In contrast, *Il1β* showed a sexually dimorphic methylation pattern, but these differences were not attributable to immune stimulation.

**Discussion:**

The results support the presence of an epigenetic interplay between sex and immune response in the fish gonads. Sex-dependent methylation changes in *Casp9* following LPS exposure, together with the inherent sexual dimorphism observed in *Il1β*, indicate that immune stimulation and sex jointly shape epigenetic landscapes of innate immune genes in reproductive tissues. Although the feminization effect was not statistically significant, the observed trend suggests that immune activation during the critical gonadal differentiation window may influence sex outcomes. Overall, these findings contribute to a deeper understanding of the epigenetic mechanisms underlying sexually dimorphic immune responses in reproductive tissues and highlight important avenues for future research.

## Introduction

The Rauta et al. (2012) innate immune response is the first line of defense, recognized for its nonspecific nature, meaning it does not require prior exposure to the invader’s surface markers (Tort et al., 2003). Press and Evensen (1999) interleukins, a broad group of cytokines, play a vital role in immune defense by attracting leukocytes to infection sites to trigger inflammation, while caspases are a family of protease enzymes crucial for apoptosis and the function of the inflammasome (McIlwain et al., 2013). Interleukin 1 beta (*Il1β*) is critical in the early stages of the inflammatory response. After being cleaved into its active form by caspase (*Casp9*), *Il1β* binds to its receptor, which then activates the nuclear factor NF-κB pathway (Brocker et al., 2010). Specifically, *Casp9* plays a key role in the mitochondria-driven cell death pathway (Shalini et al., 2015).

Interactions between the immune and reproductive systems have been observed in fish, with studies indicating that immune challenges can impact reproductive health and *vice versa* (Encel et al., 2023; Krams et al., 2017; Schreck, 2010; van Gelderen et al., 2024). It has also been shown that the immune system response capacity is closely linked to sexual dimorphism (Caballero-Huertas et al., 2024; Campbell et al., 2021). This close relationship between systems could be more compromised in those species with sexual differentiation dependent on the environment. To date, only a couple of studies have revealed that immune stimulations during gonadal differentiation at the larval stage were able to alter sex ratios towards females. Results showed that heat-killed *Escherichia coli* were able to activate the NF-κB pathway, which mediates apoptotic routes, affecting gonad differentiation and resulting in ovarian development from 30% to 85% of the final population in zebrafish (*Danio rerio*) (Moraleda-Prados et al., 2021; Pradhan et al., 2012). More recent research revealed that feminization was dependent on a dose and strain of the lipopolysaccharide (LPS) used during sex differentiation in the same fish species (Moraleda-Prados et al., 2021).

Elevated temperatures can lead to the masculinization of fish populations, a phenomenon with significant consequences for aquaculture but also in a climate change scenario. This effect is particularly relevant for those species with clear sexual dimorphism, like the European sea bass (*Dicentrarchus labrax*), for which farmed females are preferred due to their faster growth and larger size compared to males (Piferrer et al., 2005; Saillant et al., 2002). Additionally, rising ocean temperatures caused by climate change have profound effects on fish physiology and evolutionary processes that might alter the final phenotype (Geffroy et al., 2020). Given the evidence suggesting that immune stimulation may promote feminization, there is an opportunity to investigate immune-based treatments during sex differentiation to increase the proportion of females in farmed populations. Therefore, aiming to develop an *in vivo* model to modulate sex ratios toward females could ultimately enhance fish production efficiency and lead to a better understanding of our ecosystem.

Epigenetics, the study of heritable changes in gene expression that do not alter the underlying DNA sequence, has emerged as crucial in understanding various biological processes. DNA methylation, a well-characterized epigenetic modification, involves adding a methyl group to cytosine residues, typically regulating gene activity by modulating chromatin structure and transcription factor binding (Bird, 2002). In mammals and fish, epigenetic responses are implicated in the infection process, with pathogens inducing host DNA methylation changes to evade immune responses (Gómez-Díaz et al., 2012). The interaction between epigenetics and the immune system is known, although not deeply studied, particularly in fish. It was observed by studying the two innate immune-related genes, i.e., *Casp9* and *Il1β,* in zebrafish larvae that they were significantly hypomethylated after LPS treatment (Moraleda-Prados et al., 2021). Thus, these genes may have a major role in the cascade of sexual differentiation after LPS immunostimulation in zebrafish larvae. However, to date, the DNA methylation pattern of key innate immune genes in the adult gonads after immunostimulation has never been elucidated.

With multiple applications ranging from biomedicine to aquaculture production, the zebrafish is an optimal model for understanding immune aspects better (Dixon et al., 2006; Gonzalez-Covarrubias et al., 2022; Piferrer and Ribas, 2020; Ribas and Piferrer, 2014; Trede et al., 2004). Zebrafish has been a model to study host-pathogen relationships, disease resistance, innate and acquired immune responses, treatments against pathogens, as well as the genetic and epigenetic mechanisms involved in these processes (Dixon et al., 2006; Trede et al., 2004). The aims of this study were, on one hand, to examine sex ratio alterations following LPS stimulation during sex differentiation during larva development to establish a reliable *in vivo* model to modulate the final phenotype toward females through immune stimulation. On the other hand, the study aimed to investigate in detail the DNA methylation patterns of the two key immune markers in the adult gonads after immune stimulation. Based on existing literature, we hypothesized that: (1) immunostimulation via immersion may alter the expected 1:1 sex ratio by increasing the proportion of females in the final population; (2) the immune genes *Casp9* and *IL1β* exhibit distinct methylation patterns in the gonads after immune stimulation, potentially leading to different sexual responses between females and males. In this study, we aimed to deepen our understanding of phenotypic changes resulting from environmental factors (i.e., immune system activation) on gonadal development. Additionally, we sought to elucidate the epigenetic regulation of immune system genes in fish within the context of reproduction and immunity, potentially uncovering novel mechanisms that link these two critical systems.

## Materials and methods

### Animal rearing conditions

The TUE zebrafish strain used in our study was housed in the animal facilities of the experimental aquariums at the Institute of Marine Sciences (ICM-CSIC) in Barcelona, Spain. The zebrafish were raised in a custom-built, self-contained recirculating system with a water flow rate of 3,000 L per hour, equipped with a UV light system to effectively eliminate any bacteria carried by the water flow effectively. This system was located in a controlled environment chamber with a 12-h light and 12-h dark photoperiod, maintaining an air temperature of 26 °C ± 1 °C and a humidity level of 60% ± 3%.

We closely monitored various physicochemical parameters daily, as per the guidelines established by Ribas and Piferrer (2014). These parameters included maintaining the water temperature at 28 °C ± 0.2 °C, a pH level of 7.2 ± 0.5, conductivity within the range of 750–900 μS, and dissolved oxygen levels between 6.5 and 7.0 mg per liter. Quality parameters such as sulfite, sulfate, nitrate, and ammonia were assessed weekly using commercial testing kits, and periodic assessments were conducted by the water analysis service at ICM-CSIC to ensure the highest water quality standards.

For natural egg fertilization, a single female and a male from different family lines were placed together in mating tanks to preserve interfamily genetic diversity, a key characteristic observed in this fish species (Liew et al., 2012; Ribas et al., 2017; Ribas et al., 2017). The total number of fertilized eggs was recorded to ensure fertility within the reference values established for this species by Ribas and Piferrer (2014). Furthermore, the survival of the fish post-hatch was in accordance with the guidelines set by the OECD for the Fish Sexual Development Test (OECD, 2011).

A total of two biological replicates from different independent families were used. Approximately 50 eggs were arranged in each Petri dish containing E3 embryonic medium (with a pH of 7.2 ± 0.5) and supplemented with 0.1% methylene blue (Sigma-Aldrich, Madrid, Spain). These dishes were incubated at a stable temperature of 26 °C ± 1 °C until 6 days post-fertilization (dpf). Subsequently, larvae were transferred into 2.8-L plastic tanks (Aquaneering, ZT280). To avoid unwanted masculinization due to elevated rearing density, the number of fish per tank was kept in the range of 25–35, based on our previous study of the effects of density on zebrafish sex ratios (Ribas et al., 2017).

Feeding regimens were tailored to the developmental stages of the fish. From 6 to 15 dpf, the fish were provided with specialized dry larval food (17. FO.CE.0611, Sparos I&D Nutrition in Aquaculture). Starting from 15 dpf and as they continued to grow, the fish were transitioned to pellets (AquaSchwarz, Göttingen, Germany), with the pellet size adjusted to accommodate their increasing size. Throughout all stages up to 10 dpf, the diets were supplemented with Artemia nauplii (AF48, INVE Aquaculture, Dendermonde, Belgium).

### Immune stimulations during sex differentiation to determine the sex ratio

At 17 dpf, the fish from two families were randomly divided into three groups: control (CTRL), LPS, and SHAM—with an equal number of fish as previously described by Moraleda-Prados et al. (2021). Briefly, the larvae of each group were subdivided into 20 larvae per tank. The LPS and CTRL groups consisted of a minimum of 60 larvae in total (three technical replicates), and the SHAM group consisted of a minimum of 40 larvae (two technical replicates). The sham group remained in the system throughout the entire experiment to serve as a stress control for fish handling and manipulation.

For the immune stimulation baths of the CTRL and LPS groups, 12 mL glass bottles were used. Each bottle consisted of 20 fish and a final volume of 12 mL. The CTRL group consisted of only tank water, while the LPS group consisted of tank water and LPS from *Pseudomonas aeruginosa* with a concentration of 20 μg∙mL^−1^. The SHAM group was used as a stress control. To determine the treatment concentration, previous pilot studies were performed (Moraleda-Prados et al., 2021). The larvae were kept in an incubator (28 °C) for 30 h. During these 30 h, the larvae were not fed.

After 30 h, the larvae were washed to remove the LPS. Washing was done by placing the larvae in a strainer into a breeding tank filled with tank water (28 °C). After 5 min, the strainer with the larvae was placed into a new breeding tank with fresh tank water. This process was repeated a total of 3 times and was performed for both the LPS group and the control group. After washing, the larvae were put back in the correct tank and fed with Artemia. The LPS bath/LPS wash was performed twice a week, with a 24 h rest period between each LPS bath/LPS wash.

Immune stimulation baths were performed a total of 6 times over 3 weeks. After the bath treatments, fish survival was periodically monitored during the 90 days. Afterwards, the adult fish were sacrificed by cold thermal shock, and the sex of the fish was visually assessed under the microscope.

### Immune stimulation experiments in adult fish to study DNA methylation alterations

Adult male and female zebrafish (180 dpf, mean weight 0.24 ± 0.06 g and mean standard length, SL, of 2.51 ± 0.28 cm) were divided into two groups: a control group and an immune-stimulated group with *P. aeruginosa* LPS prepared according to the manufacturer’s instructions (Sigma-Aldrich, MFCD00131520). LPS was diluted with MQ water at a concentration of 100 μg/mL. To determine the treatment concentration, previous pilot studies were performed (Moraleda-Prados et al., 2021).

Fish were anesthetized with 100 mg∙mL^−1^ of tricaine methanesulfonate (MS222, Sigma) for 10 min before intraperitoneal (IP) injections. Fish were injected either with LPS (2 μg, volume of 2 μL) for the treated group or phosphate buffered saline (PBS) for the control group (van Gelderen et al., 2023). After IP injections, fish were returned to the corresponding tanks to recover. At 24 h post IP injection, fish were euthanized in ice-cold water followed by decapitation. The mean total weight (precision ±0.05 g) and SL (precision ±0.01 cm) were recorded. Under a magnifying glass, gonads were dissected and flash-frozen in liquid nitrogen at −80 °C until further analysis. All the gonads used for this study were mature and differentiated based on morphological characteristics as described previously (Ribas et al., 2017).

### DNA methylation analysis

Methylation levels of gonads treated for 24 h with *P. aeruginosa* LPS were studied for *Ilβ* and *Casp9* by the MBS technique following the procedures described elsewhere (Anastasiadi et al., 2018; Caballero-Huertas et al., 2020). Briefly, genomic DNA was extracted from 42 fish (Control group: N = 6 females, N = 14 males; LPS injected group: N = 8 females, N = 14 males). DNA from gonadal tissues was digested using 1 μg of proteinase K (Sigma-Aldrich, St. Louis, Missouri) overnight at 65 °C. The next day, a standard phenol-chloroform-isoamyl alcohol protocol, in conjunction with ribonuclease A (PureLink RNase A, Life Technologies), was employed, followed by bisulfite conversion by EZ DNA Methylation-Gold Kit (Zymo Research) to deaminate unmethylated cytosines, enabling downstream methylation analysis. The specific regions of interest in the gene promoters, including the promoter regions, first exons, and first introns where possible, were amplified through two rounds of PCR using custom-designed primers for the three immune genes described in (Caballero-Huertas et al., 2020; Moraleda-Prados et al., 2021). Prior to this study, the primer specificities were verified through Sanger sequencing of amplicons from a sample pool. Adaptor sequences for 16S metagenomic library preparation (Illumina) were added to the 5′ ends of these primers: Forward: TCGTCGGCAGCGTCAGATGTGTATAAGAGACAG, and Reverse: GTCTCGTGGGCTCGGAGATGTGTATAAGAGACAG. This allowed for the enrichment of CpG sites in the target regions, which included nine CpGs for *Il1β*, and 19 for *Casp9*.

#### MiSeq sequencing

The resulting PCR products were indexed using the Nextera XT Index Kit Set A (Illumina; FC-131-2001), following Illumina’s protocol for 16S metagenomic library preparation. PCR product quality was successful in 39 samples. The indexed samples were pooled in an equimolar fashion to create a single multiplexed library. Paired-end sequencing was performed on the Illumina MiSeq platform (Illumina, San Diego, California) according to the manufacturer’s protocol for paired-end sequencing (2 × 301 bp) at the National Center of Genomic Analysis (CNAG, Barcelona). Image analysis, base calling, and quality scoring were carried out with the manufacturer’s Real-Time Analysis software (RTA v1.18.54), followed by the generation of FASTQ sequence files.

### Bioinformatics analysis

Raw sequencing data were demultiplexed based on index codes using Illumina software, and adapter sequences were removed with Trim Galore! software (v. 0.4.5) (Babraham Bioinformatics). Quality control checks were performed both before and after trimming with FastQC software (v. 0.11.8) (Andrews, 2010) to verify proper adapter removal (Ewels et al., 2016). Low-quality bases (Phred score <20) were filtered out, and only paired-end (PE) reads were included in the analysis.

An *in silico* bisulfite-converted zebrafish genome (danRer11, GRCz11, GCA_000002035.4) was used as the reference for alignment. Data processing, labeling, and tabulation were conducted using the gemBS pipeline version 4.1.5. (Merkel et al., 2019). The alignment was performed using a two-step procedure with Bismark software (v. 20.0) (Krueger and Andrews, 2011). Bisulfite conversion efficiency was calculated for each sample, ensuring a minimum threshold of 99.0%, which all samples successfully met. The “BSgenome.Drerio.UCSC.danRer11” package was used to retrieve the coordinate positions of all CpG sites (TBD, 2019). Raw data were submitted to European Nucleotide Archive (ENA) with the accession number ERP183568.

Reads with MAPQ scores <20 and read pairs mapping to the same start and end points on the genome were filtered out after the alignment step. The first five bases from each read were trimmed before the variant and methylation calling step to avoid artifacts due to end repair. For each sample, CpG sites were selected where both bases were called with a Phred score of at least 20, corresponding to an estimated genotype error level of ≤1%, and where the total number of reads informative for methylation from both strands combined was ≥6. Sequencing depth, mapping rate and coverage for amplicon regions can be found in Supplementary Table S1. Coverage filtering was applied by removing CpG sites with less than ten reads.

#### DNA methylation levels

DNA methylation levels were calculated by averaging the methylation values of CpG sites within each gene for each sample and then averaging them across treatments. For individual CpG sites, methylation values were averaged by their coordinate positions for each treatment. For each gene, methylation values across covered CpG sites were averaged per individual. Sample metadata (sex and treatment condition) were compiled and linked to the methylation dataset. To formally test the effects of sex and treatment, linear models were fitted with gene-specific methylation as the response variable and sex, treatment condition (e.g., LPS stimulation), and their interaction as explanatory factors. We computed for every CpG in the two targeted loci (*Il1β* and *Casp9*), we fit an OLS model to methylation β values: β∼condition × sex (Supplementary Table S2). To visualize methylation variation between experimental groups, gene-level values were summarized and plotted as boxplots using the ggplot2 R package.

CpG location (exon, intron, promoter) and whether it overlaps transcription factor binding sites (TFBS) was analysed. We defined the promoter as ±2,000 bp around the transcript start site (TSS) for any refGene transcript of the locus; exons were taken directly from the GTF; introns were computed as transcript span minus exons; anything else was considered intergenic. Under this definition, all CpGs probed in both *Il1β* and *Casp9* fall within the merged promoter region of their respective genes (Supplementary Tables S3 and S4). For TFBS in *Casp9*, we intersected CpG intervals (1–2 bp windows) using the UCSC JASPAR TFBS table. Overlaps were reported per CpG together with the list of TF names that cover that CpG. For *Il1β*, we repeated the intersection using the UCSC JASPAR TFBS table; only CpGs whose 1–2 bp interval overlaps a motif window are reported as TFBS-positive (Supplementary Tables S3 and S4).

### Statistical analysis of fish survival and sex ratio

For determining survival, the Kolmogorov–Smirnov and Levene’s tests were used to check data normality and the homoscedasticity of variances, respectively. Then, a one-way analysis of variance (ANOVA) was used to detect possible differences among groups. Tukey’s test was used to perform *post hoc* multiple comparisons. Model diagnostics and summary statistics were generated in R.

## Results

### Fish survival

**FIGURE 1:**
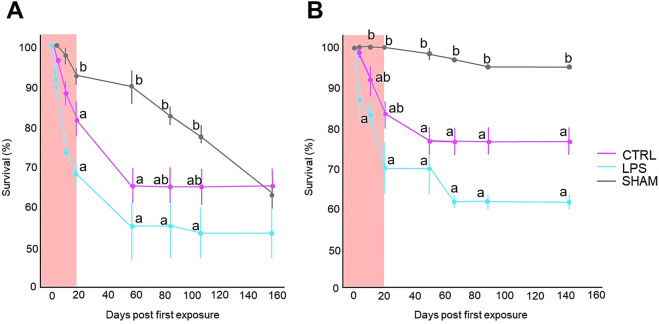
Percentage (%) of larval survival from the two experiments conducted for Family 1 **(A)** and Family 2 **(B)** following immune stimulation during sex differentiation. Larvae from *Pseudomonas aeruginosa* group were exposed to a concentration of 20 μg∙mL^−1^. Only water was used for the control group. SHAM group grew normally without any handling. The larvae were kept in an incubator (28 °C) for 30 h during the LPS treatments in which larvae were not fed. The LPS bath/LPS wash was performed twice a week, with a 24 h rest period between each LPS bath/LPS wash. Immune stimulation baths were performed a total of 6 times for 3 weeks. Data show the mean ± SEM. Statistically significant differences (*P* < 0.05) against the control group per time lapse are indicated by different letters and were examined by one-way analysis of variance (ANOVA), and Tukey’s test was used to perform *post hoc* multiple comparisons.

Survival was tested by counting the number of fish in the control, LPS, and SHAM groups during the experiment. As reflected in Figure 1, the survival rate of the LPS group was lower than that of the CTRL and SHAM groups. Specifically, the survival of all groups at the first test (Family 1), reflected by Figure 1A, differed significantly among them (*P* < 0.05), while in the second test (Family 2) (Figure 1B), only significant differences were found between the control and SHAM groups. There were no extreme declines in the number of fish in any of the groups.

### Sex ratio analysis

**FIGURE 2:**
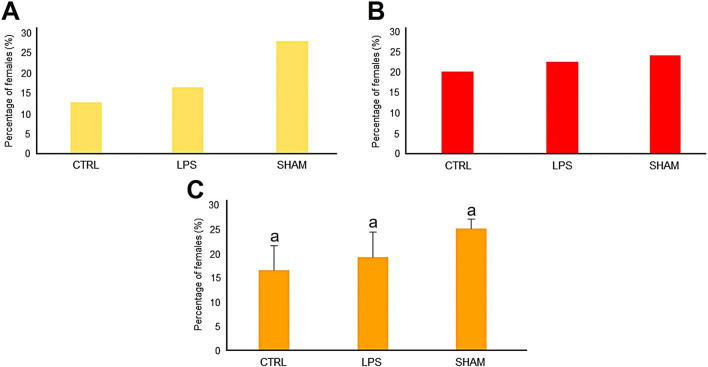
Sex ratio results of treated zebrafish larvae with *Pseudomonas aeruginosa* LPS bath experiments of family 1 **(A)** and family 2 **(B)** for 3 weeks. **(C)** Combined results from the two experimental families.

The percentage of females for Family 1 for the CTRL, LPS, and SHAM groups was 13%, 16%, and 28%, respectively (Figure 2A), while for Family 2 it was 20%, 23%, and 24%, respectively (Figure 2B). The sex ratio for the two families together resulted in percentages of females of 17%, 19%, and 25%, respectively (Figure 2C). All data showed a feminization trend, although there was no significant difference in the number of females between treatment groups.

### DNA methylation patterns

The total number of sequencing reads was 73,695,800 and 46,242,693 reads were mapped on to the zebrafish genome, with a 79.75% of mapping to the studied amplicons. All the samples passed the sequencing high-quality scores.

**FIGURE 3:**
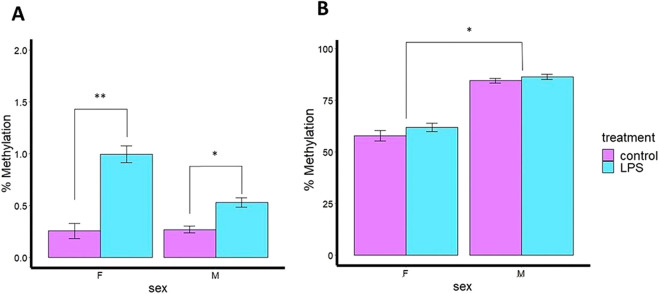
Percent (%) of DNA methylation levels of immune genes of 180 days post fertilization (dpf) zebrafish intraperitoneally injected (IP) with *Pseudomonas aeruginosa* LPS in gonads of adult zebrafish. **(A)** *Casp9* percentage of mean DNA methylation levels after *P. aeruginosa* LPS injection. **(B)** *Il1β* percentage of mean DNA methylation levels after *P. aeruginosa* LPS injection. F: female; M: male.

Although the percentage of methylation was very low for *Casp9* gene (∼1.5%), significant differences in *Casp9* DNA methylation were observed between the control and LPS groups in both ovaries (*P* < 0.01) and testes (*P* < 0.05) of adult fish 24 h after intraperitoneal (IP) injection with LPS indicating that *Casp9* methylation is sensitive to LPS treatment (immune challenge), with subtle sex differences (Figure 3A). Ovaries treated with LPS exhibited a 2-fold increase in DNA methylation (hypermethylation) of *Casp9* compared to the CTRL group, similar to that observed in testes, showing that *Casp9* methylation is driven by LPS stimulation, with some sex dependence.

In contrast, *Il1β* did not show significant differences (*P* > 0.05) in DNA methylation due to LPS treatment. However, significant differences (*P* < 0.05) were observed related to sexual dimorphism (Figure 3B), in which testes had 26% higher DNA methylation than ovaries. The data indicate that *Il1β* DNA methylation is sex-dependent in gonads, regardless of immune stimulation.

**FIGURE 4:**
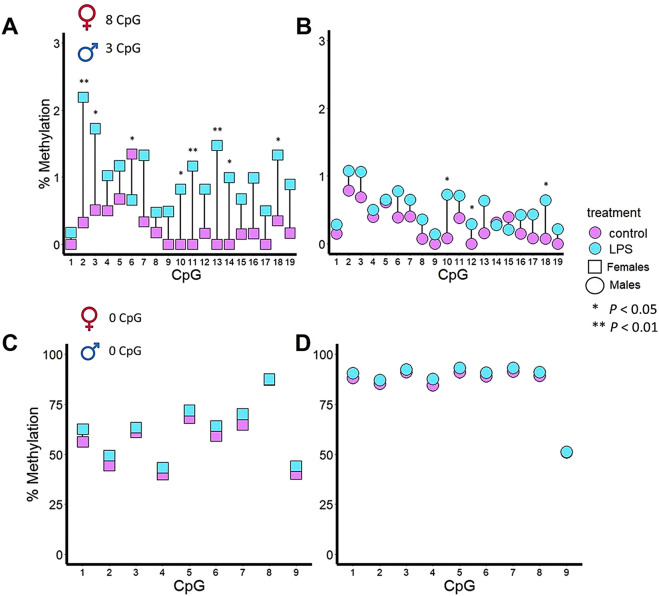
DNA methylation patterns of two innate immune genes in the gonads of zebrafish after 48 h of immune stimulation by LPS. *Casp9* differences in DNA methylation percentage for each of the 19 CpG analyzed in ovaries **(A)** and testes **(B)** between control and IP fish gonads. A total of 8 CpG and 3 CpG were significant in ovaries and testes, respectively. *Il1β* differences in DNA methylation percentage for each of the 9 CpG analyzed in ovaries **(C)** and testes **(D)** between control and IP fish gonads. No CpGs in ovaries or testes differed significantly between the control and the treated group.

In ovarian tissues, *Casp9* showed, at individual CpG sites, a significant difference in DNA methylation in eight of the 19 CpG sites (Figure 4A), i.e., CpG2 (*P* < 0.01), CpG3, (*P* < 0.05), CpG6 (*P* < 0.05), CpG10 (*P* < 0.05), CpG11 (*P* < 0.01), CpG13 (*P* < 0.01), CpG14 (*P* < 0.05) and, CpG18 (*P* < 0.05). In testicular tissues, *Casp9* showed, at individual CpG sites, a significant difference in DNA methylation in three of the 19 CpG sites (Figure 4B), i.e., CpG10 (*P* < 0.05), CpG12 (*P* < 0.05) and CpG18 (*P* < 0.05). Thus, CpG10 and CpG18 were hypermethylated in both sexes after LPS IP. In the DNA methylation pattern of *Il1β* CpG sites, no significant changes were observed after 24 h of LPS treatment in ovaries (Figure 4C) or testes (Figure 4D).

From the TFBS analysis we found that For IL1B, CpG8 and CpG10 exhibited overlap with multiple transcription factor binding sites (TFBS). CpG8 coincided with motifs for *eomes, tbx2,* and *tbx20*, whereas CpG10 displayed a high density of TFBS, including binding motifs for *atoh7, ets2, fos::jun, foxo4, runx2,* and members of the tead family. The presence of these factors, many of which are implicated in immune signaling and stress responses, highlights the potential functional relevance of these CpGs in transcriptional regulation.

Similarly, in *Casp9*, CpG10 was located within the promoter region and overlapped with TFBS for transcription factors such as *ascl1, atoh7, mxi1, nr2f2,* and *snai1*. These factors are associated with developmental pathways and cellular stress responses, suggesting that CpG10 may serve as an epigenetic node influencing apoptotic signaling under immune challenge.

## Discussion

This study provides novel insights into the sexually dimorphic epigenetic regulation of innate immune genes in zebrafish gonads following immune stimulation. By integrating developmental and adult-stage immune challenges with high-resolution DNA methylation profiling, we demonstrated that immune activation via LPS induces gene- and sex-specific epigenetic modifications, particularly in *Casp9*, while *Il1β* exhibits stable sexual dimorphism independent of treatment. Although no statistically significant shifts in sex ratios were observed following LPS immersion during gonadal differentiation in early development, the consistent trend toward feminization aligns with previous findings in zebrafish and other teleosts (Caballero-Huertas et al., 2020; Pradhan et al., 2012). The three studies suggest that immune activation during the sensitive period of sex differentiation may influence gonadal fate, potentially through modulation of apoptotic pathways and cytokine signaling cascades (Pradhan et al., 2012). The observed masculinization tendency of both control and LPS groups in comparison to the SHAM group further underscores the sensitivity of the sex differentiation process to environmental and handling stressors that might have occurred due to fish manipulation during the protocol. This masculinization may act through the hypothalamic-pituitary-interrenal (HPI) axis and its downstream epigenetic effectors (Castañeda Cortes and Fernandino, 2020; Castaneda Cortes et al., 2019). In a previous study, we found differences in the response to the LPS strain and concentrations, i.e., *Aeromonas hydrophila*, 75 μg/mL LPS, during sex differentiation in the same fish species (Moraleda-Prados et al., 2021). Thus, the developmental period of the treatment, the strain, the dose and even the fish genetic background might be relevant for the significant sex ratio bias towards females. Nevertheless, more research in this direction should be performed.

To our knowledge, this is the first time that DNA methylation patterns of two innate immune genes have been studied in the fish gonads after immunostimulation. In the context of immune-reproductive interaction, a previous study elucidated the sexual dimorphism in the DNA methylation levels of fish gonads at 90 dpf for the genes *Casp9* and *Il1β* in zebrafish (Caballero-Huertas et al., 2020). On the other hand, after immunostimulation, changes in the methylation of these two genes were observed in larvae during gonadal development (Moraleda-Prados et al., 2021). Aiming to shed light on the influences of the immune system on the gonadal development, we combined studies of immunostimulation during gonadal development and its effect on DNA methylation patterns in the ovaries and testes in adult fish to better understand the immune mechanisms and their sexually dimorphic influence. In this context, the present study shows that *Il1β* is more methylated in testes, while methylation in *Casp9* is similar in both gonadal types. However, LPS treatment seems to increase the methylation of *Casp9* in both sexes, but is more noticeable in females while *Il1β* DNA methylation is higher in testes. Within this line, metabolomic analysis in zebrafish gonads revealed that testes altered the immune system 48 h after LPS stimulation (van Gelderen et al., 2023). In a recent study on European sea bass infected with bacteria, sexually dimorphic patterns were observed in the metabolites released in the gonads. For example, uric acid was differentially regulated—being inhibited in one sex and released in the other (Caballero-Huertas et al., 2025). In addition, testes showed a greater number of genes altered in the transcriptome, together with microRNAs, compared to ovaries in the European sea bass after infections (van Gelderen et al., 2024), indicating that the transcriptome and miRNome show a clear sexual dimorphic pattern when coping with a challenge. These studies reveal that molecular pathways are altered at multiple levels—from epigenetic regulation to metabolite release in gonadal cells—following an immune challenge in fish. This supports the existence of a cross-talk between the immune and reproductive systems, mediated by epigenetic mechanisms.

The analysis of methylation, specifically, of CpGs, is essential to decipher the concrete regions of the genes that are susceptible to being modified by epigenetic mechanisms. DNA methylation alterations of specific CpGs are sensitive to environmental influences and can be associated with a particular phenotypic response (Beemelmanns et al., 2021). Epigenetic biomarkers (epibiomarkers) are determined during the early developmental stage. Due to their sensitive response, they have been used in age assessment, sex prediction, and the effects of the environment on fish (Anastasiadi and Piferrer, 2023; Zhang et al., 2023). In mammals, CpG methylation is associated with sexually dimorphic expression of sex steroid hormone-related genes in conjunction with histone modification, while in fish, only a few studies have discussed the influence of CpG methylation of sex-related gene promoters on gene expression patterns (Wen et al., 2014).

The *Casp9* gene, which is shown to be key in the mitochondria-mediated cell death pathway (Sakamaki and Satou, 2009), showed significantly higher DNA methylation in both immunostimulated sexes. This could be observed in several CpG sites in both gonadal types, although more marked in females. In this line, *in silico* associations between transcriptomic data obtained after *A*. *aeruginosa* LPS challenge with methylation data obtained after chronic stress exposure showed that some immune genes, including *Casp3a*, were hypermethylated and reduced their expression in Atlantic salmon fry (Uren Webster et al., 2018). In contrast, *Casp9* expression in the testis was gradually enhanced after starvation (Fan et al., 2021), indicating that the expression levels of *Casp9* in the testis depend on the stressor. In our study, *Casp9*, even though a general increase of methylation was seen, different mechanisms were triggered after exposure to the immunostimulatory agent in the two sexual tissues, coinciding with hypermethylation of CpG 10 and 18. This could mean that these CpGs are intrinsically susceptible to being altered by epigenetic mechanisms, while CpGs 2, 3, 6, 11, 13, and 14 for the ovaries, and 12 for the testes, are exclusive to each sex, which is of great interest for the development of epibiomarkers. Nevertheless, further experimental validation is required to determine whether the methylation status of these CpG sites can reliably serve as epigenetic biomarkers for sex differentiation in teleost species.

The exclusive localization of CpG sites within promoter regions and their overlap with multiple TFBS underscores the regulatory potential of these loci in Il1*β* and Casp9. In Il1*β*, the enrichment of TFBS for factors involved in inflammatory signaling (e.g., *fos::jun, ets2*) suggests that these CpGs may act as critical hubs for transcriptional control, even in the absence of methylation changes under acute stimulation. Conversely, the TFBS landscape at CpG10 in Casp9, featuring regulators such as *snai1* and *atoh7*, points to a possible epigenetic mechanism modulating apoptotic pathways during immune stress (Hollenhorst et al., 2011; Peinado et al., 2007; Shaulian and Karin, 2002). These observations support the concept that epigenetic regulation is not solely determined by global methylation levels but also by the spatial interplay between CpG sites and TFBS within promoter regions (Schübeler, 2015; Yin et al., 2017). Future integrative studies combining methylome, transcriptome, and chromatin accessibility data will be essential to elucidate the functional consequences of these interactions.

Methylation in cytokine *Il1β* was not significantly affected by LPS in any of the targeted tissues. However, an underlying sexual dimorphism was observed for this gene that was not observed with *Casp9*. The DNA methylation pattern of *Il1β* in fish is not well addressed. However, it is known that *Il1β* changes the expression in the fish gonads. In sea bream (*Sparus aurata*) gonads, *Il1β* was found in the cytoplasm of spermatogonia, spermatocytes, and oogonia, with a higher expression during reproductive cycles (Chaves-Pozo et al., 2008). *Il1β*, together with cytokine *TNFα*, has roles in regulating testicular androgen production in goldfish (Lister and Van Der Kraak, 2002). Further, stress caused by starvation altered the expression of *Il1β* and other cytokines were consistently elevated in zebrafish (Fan et al., 2021). Also, our previous study in zebrafish showed a marked dimorphism both in methylation (hypermethylation in testes) and gene expression (overexpression in male gonads) (Caballero-Huertas et al., 2020). However, neither global methylation nor CpGs modifications by immunostimulation seemed to alter its potential expression in this study. The lower methylation levels found could be explained by the experimental approach to immunostimulation. Both bath and IP seem effective as immune stimulation methods (Crosbie and Nowak, 2004; Evans et al., 2004; Ispir and Dorucu, 2014; Toranzo et al., 1995), even though IP provides more antibody activity and protection. However, it must be considered that to carry out generalized treatments on a stock of fish, and thus simulate rearing strategies, immunostimulation by immersion is faster and does not require direct manipulation of the individuals. Also, it should be considered that the dynamics of infection may differ between different pathogens or infection systems (Foott, 2025). By immersion, it resulted in an initial attachment of the bacteria to the epithelia, after which there is a gradual shift from the surface to the swim bladder and blood, and finally to internal tissues, whereas IP resulted in an acute systemic infection that proceeded to extend to the hypodermis and peritoneal cavity, followed by internal tissues (Schmidt et al., 2017).

The discrepancies between methylation patterns observed in LPS-treated larvae (whole-body analysis) in our previous study (Caballero-Huertas et al., 2020; Moraleda-Prados et al., 2021) and adult LPS-treated gonads (tissue-specific analysis) underscore the importance of spatial resolution in epigenetic studies. In larvae, immune stimulation induced hypomethylation of *Casp9*, whereas in adult gonads, hypermethylation was predominant. This divergence likely reflects the tissue-specific functions of immune genes and the dynamic nature of epigenetic regulation during development. This is because genes may contribute to tissue-specific functions (Blake et al., 2020), and in the gonads, immune gene function seems to be diversified not only in the role of innate defense but also in sexual differentiation and the maintenance, homeostasis, and reproductive developmental cycle (Fan et al., 2021; Pradhan et al., 2012). Future studies employing single-cell methylome and transcriptome profiling will be essential to disentangle cell-type-specific responses and to map the epigenetic landscape of gonadal differentiation with higher precision.

## Conclusion

This study provides the first evidence of sexually dimorphic DNA methylation responses in innate immune genes within the gonads of zebrafish following immune stimulation. The differential methylation of *Casp9* in response to LPS, particularly the sex-specific CpG sites, underscores the epigenetic plasticity of immune genes and their potential role in mediating immune-reproductive cross-talk. In contrast, the stable yet sexually dimorphic methylation of *Il1β* suggests a developmentally programmed regulation that may contribute to sex-specific immune competence. Although the feminization trend observed after LPS immersion did not reach statistical significance, the molecular data support the hypothesis that immune activation during sex differentiation can influence the epigenetic landscape of key regulatory genes. These findings open new avenues for the development of epigenetic biomarkers for sex prediction and immune responsiveness in aquaculture species. Future research should focus on integrating transcriptomic and single-cell epigenomic approaches to dissect the cell-type-specific dynamics of immune gene regulation during gonadal development. Moreover, exploring the long-term phenotypic consequences of early-life immune stimulation will be essential to assess the feasibility of immune-based strategies for sex control and disease resilience in fish.

## Data Availability

The datasets presented in this study can be found in online repositories: in the European Nucleotide Archive (ENA) with the accession number ERP183568 and in the Supplementary Material.
